# Artificial intelligence in clinical medicine: catalyzing a sustainable global healthcare paradigm

**DOI:** 10.3389/frai.2023.1227091

**Published:** 2023-08-29

**Authors:** Gokul Krishnan, Shiana Singh, Monika Pathania, Siddharth Gosavi, Shuchi Abhishek, Ashwin Parchani, Minakshi Dhar

**Affiliations:** ^1^Department of Internal Medicine, Kasturba Medical College, Manipal, India; ^2^Department of Emergency Medicine, All India Institute of Medical Sciences, Rishikesh, India; ^3^Department of Geriatric Medicine, All India Institute of Medical Sciences, Rishikesh, India

**Keywords:** deep learning, machine learning, internal medicine, neural networks, clinical medicine

## Abstract

As the demand for quality healthcare increases, healthcare systems worldwide are grappling with time constraints and excessive workloads, which can compromise the quality of patient care. Artificial intelligence (AI) has emerged as a powerful tool in clinical medicine, revolutionizing various aspects of patient care and medical research. The integration of AI in clinical medicine has not only improved diagnostic accuracy and treatment outcomes, but also contributed to more efficient healthcare delivery, reduced costs, and facilitated better patient experiences. This review article provides an extensive overview of AI applications in history taking, clinical examination, imaging, therapeutics, prognosis and research. Furthermore, it highlights the critical role AI has played in transforming healthcare in developing nations.

## Introduction

Artificial Intelligence (AI) has become an increasingly important technology in today's era. AI seeks to build machines that can learn, solve problems, make decisions, and perceive in a manner similar to that of humans (IBM, 2023). It involves developing computer systems and algorithms that can process enormous volumes of data, analyse that data, and generate predictions or conclusions based on that data. Several of the most recent developments in the subject have been driven by Machine Learning (ML), a branch of AI. ML algorithms are able to analyse large datasets and learn patterns and insights that can be used to make predictions and automate complex tasks (IBM, 2023). It is important to understand certain terminologies in AI before moving forward to its applications (Table 1).

**Table 1:** Terminology in AI.

Machine learning	Involves programming algorithms to make decisions or predictions based on data
Deep learning	A type of ML that utilizes artificial neural networks for processing and analyzing a significant amount of data
Natural language processing	A branch of AI that aims to teach computers how to comprehend and analyse human language
Robotics	The study and development of robots capable of performing tasks autonomously or with human assistance
Computer vision	Machines' ability to interpret and comprehend images and video
Expert systems	AI systems that mimic a human expert's decision-making abilities in a specific field
Neural networks	Computer systems that are designed to mimic the structure and function of the human brain
Cognitive computing	AI systems that can simulate human thought processes, including perception, reasoning, and problem-solving
Natural language generation	The process of using AI to generate human-like language in written or spoken form
Data mining	The revelation of patterns and insights in large datasets
Bayesian networks	A probabilistic graphical model used for probabilistic reasoning
Swarm intelligence	The collective behavior of decentralized, self-organized systems
Decision trees	A graphical representation of decision-making processes
Support vector machines (SVM)	A machine learning algorithm used for classification and regression analysis
Human-in-the-loop	A type of AI system that incorporates human feedback to improve performance

**Figure 1:**
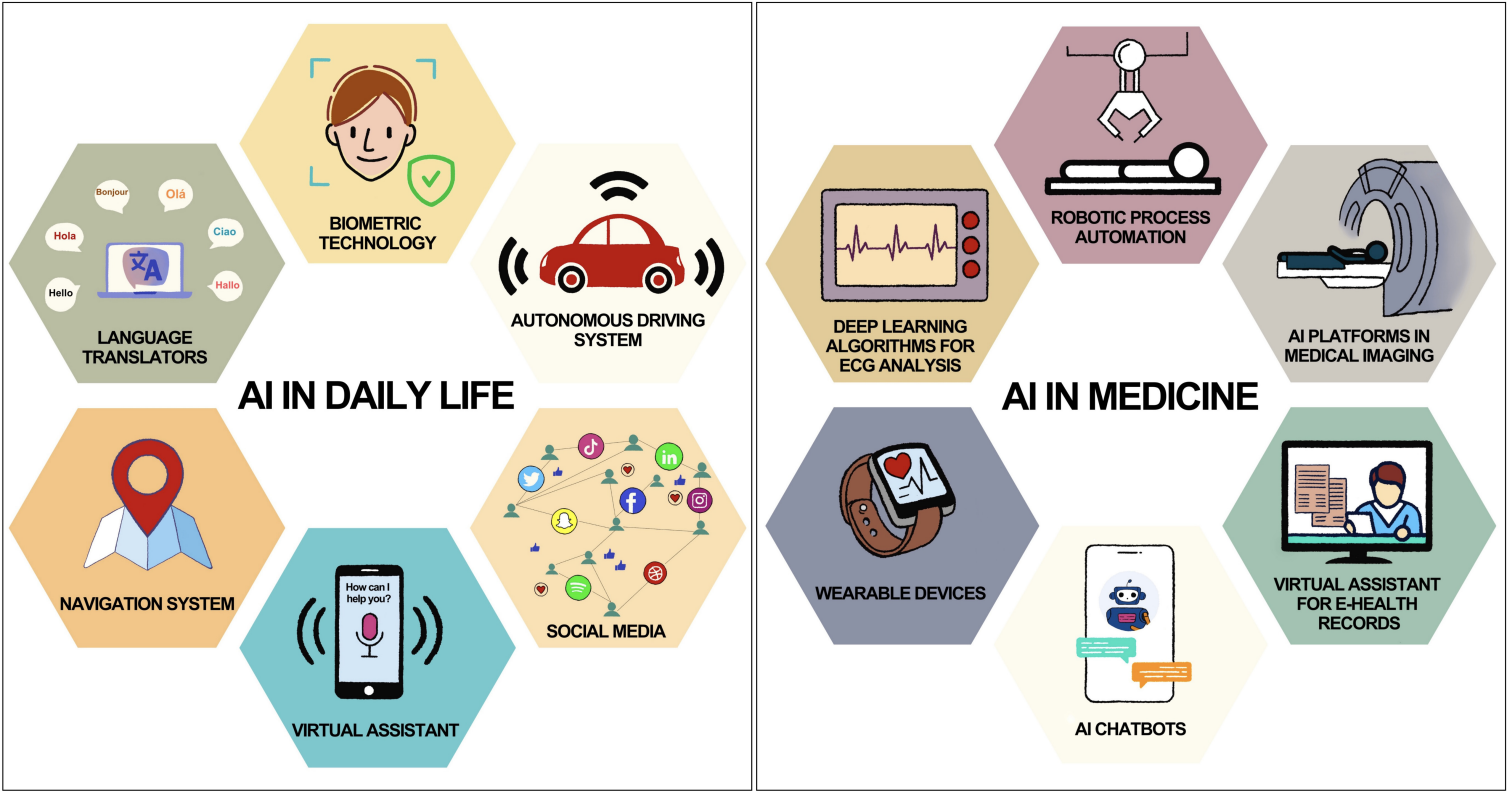
Common applications of AI in daily life and medicine.

AI is a potent technology with the potential to favorably enhance many domains of our life by automating tasks, improving decision-making, and enabling new kinds of interactions between humans and machines. Without consciously realizing it, humans are constantly surrounded and helped by AI in their daily lives (Figure 1). For example, AI-powered virtual assistants like Siri or Alexa understand natural language and respond to voice commands, making it easier for people to perform tasks like setting reminders or playing music without having to use their hands (The AI of Alexa and Siri, 2018). Navigation apps like Google Maps use AI algorithms to analyse traffic patterns and provide real-time recommendations for the fastest route (The AI of Alexa and Siri, 2018; A smoother ride and a more detailed Map thanks to AI Google, 2021; IBM, 2023). Online retailers and streaming services like Amazon, Netflix, and Spotify use AI algorithms to recommend products and content based on a user's past behavior and preferences (Netflix Recommendations, 2022; How Amazon Uses AI to Dominate Ecommerce: Top 5 Use Cases, 2023; How Spotify Uses Artificial Intelligence—and What You Can Learn from It, 2023). Even in the field of medicine, there is so much use of AI without our knowledge (Figure 1). Wearable devices like Apple Watch use AI algorithms to monitor a user's health data and provide insights into their activity levels, sleep quality, and other metrics (Healthcare—Apple Watch, 2023). The device is also capable of detecting an irregular pulse using inbuilt sensors and notify the user (Perez et al., 2019). AI is being used in electrocardiogram (ECG) reading to improve the accuracy and efficiency of diagnosis and monitoring of cardiac conditions (Attia et al., 2021). It can calculate intervals, axis, detect arrhythmias and even give a diagnosis based on ECG waveform. There are so many fascinating and lucrative applications of AI in clinical medicine which shall be discussed later in this article.

**Figure 2:**
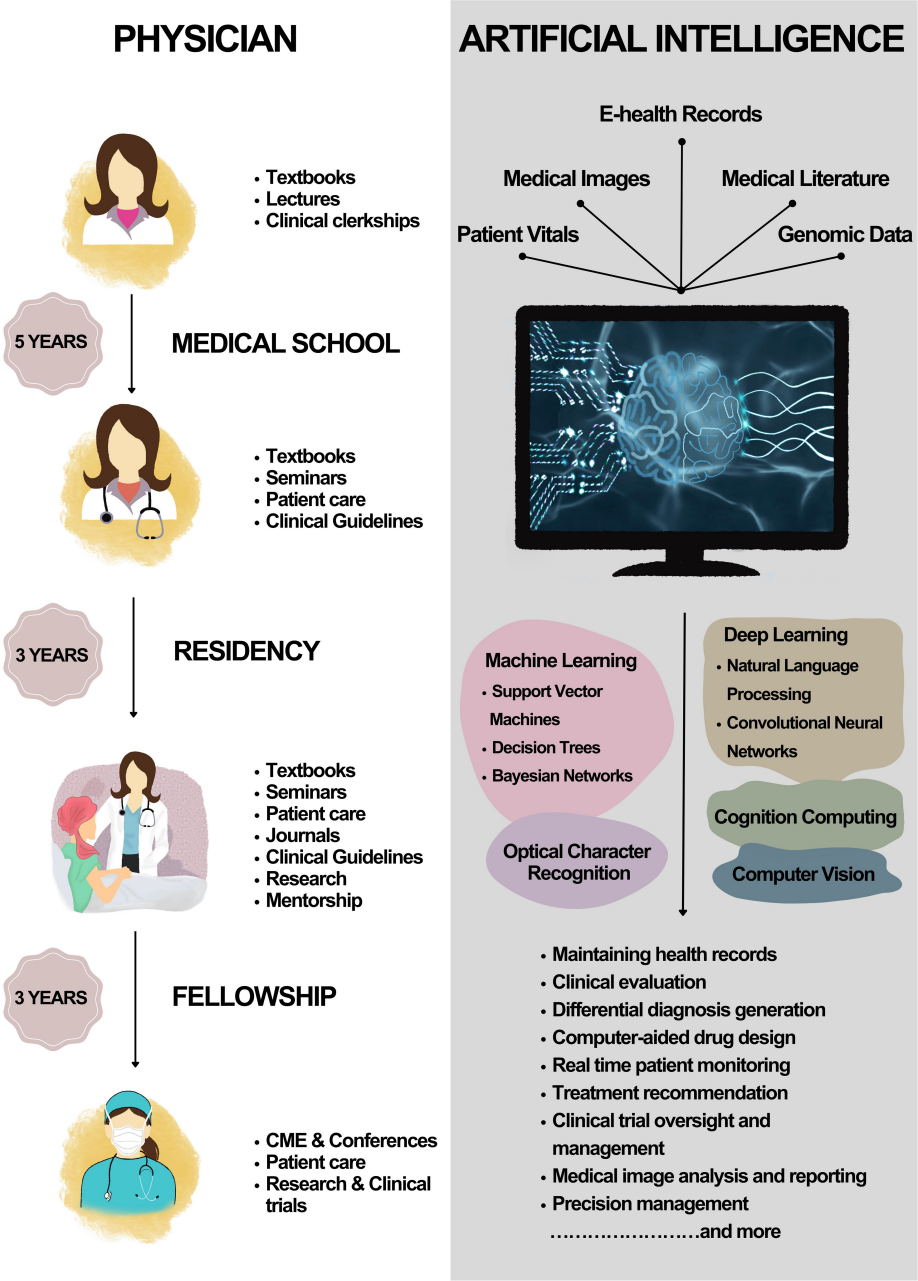
A comparison between the learning curve of a physician vs. AI.

AI-powered healthcare technology is rapidly evolving in the field of clinical medicine (Briganti and Le Moine, 2020). The integration of AI in healthcare offers significant advantages over traditional medical practice, particularly in terms of the learning curve and time efficiency. Figure 2 depicts the learning curve of a physician vs. that of AI. The learning curve is a critical factor in determining the efficiency and effectiveness of medical professionals. For medical doctors, the learning process spans years, including undergraduate education, medical school, internships, and residency. This extensive training is necessary to ensure that physicians have the knowledge and expertise to accurately diagnose and treat patients. In addition to the steep learning curve, human physicians are limited by the time it takes to evaluate and treat patients. A medical doctor can only see a finite number of patients each day, as they must spend time conducting interviews, reviewing medical records, and performing examinations. Moreover, a physician's ability to recall information and synthesize it with newly acquired data is inevitably limited by human cognitive capacity. In contrast, AI systems possess the ability to learn and adapt at an exponential rate. With access to vast amounts of data and the ability to process it in real-time, AI systems can “learn” and improve their performance through ML. This allows AI to quickly become proficient in new areas of medicine, surpassing the rate at which a human physician could acquire the same knowledge. AI systems can evaluate and treat patients with greater speed and accuracy. By processing vast amounts of medical data instantaneously, AI can generate precise diagnoses and recommend optimal treatment plans in a fraction of the time it would take a human physician. This efficiency has the potential to improve patient outcomes by allowing for more rapid intervention and reducing the likelihood of medical errors. AI systems not only have a shorter learning curve but are also capable of continuous learning and adaptation. As new medical research and discoveries emerge, AI systems can quickly integrate this information into their existing knowledge base, ensuring that they are always up-to-date with the latest advances in medicine. This adaptability allows AI to stay current and provide the most accurate and effective treatments possible, while human physicians must dedicate significant time and effort to keep pace with the ever-evolving field of medicine.

## Applications of AI in clinical medicine

### History taking

AI has the potential to significantly improve medical history taking by enabling more accurate and efficient diagnosis. Natural language processing (NLP) can be used to analyse the text or voice of a patient's responses to questions about their medical history. This can help to identify relevant information, such as symptoms, conditions, and treatments, which can then be used to create a differential diagnosis. AI-powered chatbots can provide patients with a way to answer questions about their medical history in a conversational manner (Nadarzynski et al., 2019). One of the most advanced AI chatbots available is GPT-4 (Generative Pretrained Transformer 4), a large language model (LLM) developed by OpenAI, with support from Microsoft (GPT-4, 2023). Real-time transcriptions of physician-patient conversations can be made using GPT-4 (Haug and Drazen, 2023). This can speed up the note-taking process because the doctor can examine, revise, and add to these transcriptions as needed in the future (Haug and Drazen, 2023). Another clinical intelligence solution, Nuance Dragon Ambient eXperience (DAX) product, harbors the ability to capture patient-clinician conversations and transcribe it into comprehensive clinical notes (Ambient Clinical Intelligence, 2023). ML algorithms can also be employed to assess a patient's risk for developing certain diseases based on their medical history and other factors, such as lifestyle and genetics (Kumar et al., 2022).

However, it is vital to note that chatbots have inherent limitations which requires them to be deployed thoughtfully in clinical settings (Haug and Drazen, 2023; Lee et al., 2023). They may struggle with complex histories, potentially missing out on important information. This can also be augmented by inability to detect non-verbal cues. Not to mention, the technical issues such as disruption of software and connectivity such as system crashes may pose a hindrance to patient interactions. Thus, human clinicians remain crucial in providing compassionate care. Another major limitation with chatbots is the need for proofreading notes transcribed by the language model for accuracy and missing information. At the end, the onus of patient care lies on the clinician and not the technology. Therefore, AI tools can assist in medical history taking by streamlining the process and improving efficiency, but require to be used carefully by clinicians in order to protect patient health and safety.

### Clinical evaluation

The single most essential tool for examining a patient is the doctor's hand. Fortunately, there is no AI tool which can replace the human touch. The next most prominent piece of clinical equipment is the stethoscope, which has been modified by AI for optimum performance and newer capabilities. The invent of digital stethoscopes have enabled the integration of AI into auscultation to improve diagnostic performance and compensate for inferior auscultation skills (Arjoune et al., 2023). AI-based supervised learning models have been applied to auscultation data to construct an algorithm to identify valvular stenotic lesions (Ghanayim et al., 2022). Lighter and less expensive models have also been proposed for detecting cardiac and pulmonary sounds (Zhang et al., 2023). Recently, a deep learning (DL) system (AI-ECG) was used to analyse single-lead ECGs obtained during clinical examination using an ECG-enabled stethoscope (Bachtiger et al., 2022). The system was found to detect heart failure with an ejection fraction of 40% or lower with high sensitivity and specificity. Due to the algorithm's connection to a stethoscope, it is now possible to diagnose low left ventricular ejection fraction in a larger population of patients just by doing an auscultatory exam.

Many other applications of AI in clinical evaluation have been proposed and developed in recent years. In a study, the identification of Parkinson's disease using voice signal features was proposed through the utilization of ML and DL approaches, with multilayer perceptron (MLP) and SVM models showing high accuracies of 98.3% and 95% respectively (Alshammri et al., 2023). Another study aimed to use AI and data mining approaches to diagnose COVID-19 through cough sounds, with the goal of reducing treatment costs and preventing the disease. Supervised Learning classification algorithms, such as Support Vector Machine, random forest, Artificial Neural Networks, Fully Connected neural network, Convolutional Neural Networks (CNN), and Long Short-Term Memory (LSTM) recurrent neural networks were employed. The models achieved an average accuracy of 83%, with LSTM reaching the highest accuracy at 95% (Askari Nasab et al., 2023).

The integration of AI in clinical examination has revolutionized the healthcare industry by providing more accurate and efficient diagnostic capabilities. By embracing these innovative tools, clinicians can harness the power of AI to transform clinical examination, ultimately leading to better patient outcomes, reduced healthcare costs, and an improved overall healthcare experience for all.

### Differential diagnosis

AI can significantly improve the process of making differential diagnosis by analyzing patient data and providing clinicians with a list of potential diagnoses along with the likelihood of each diagnosis being correct. AI models can use probability density functions to estimate the probability of an event occurring (Probability Density Function, 2019). This approach is commonly used in ML algorithms such as Bayesian networks, where a probability distribution is calculated based on previous observations and is updated as new data becomes available (An Overview of Bayesian Networks in Artificial Intelligence, 2023). Using decision trees, the probability of an event based on a set of rules or conditions (Gupta, 2017). Neural networks can also be used to estimate probabilities by training the network on a large dataset of inputs and their corresponding outputs, and then using the network to predict the probability of new inputs (Picton, 2023). The output layer of the network can be configured to produce a probability distribution over possible outcomes. Through these techniques and algorithms, AI can analyse patient data, such as medical images, lab results, and electronic health records, to identify patterns and anomalies that may indicate a particular disease or condition. This can help clinicians to consider a broader range of potential diagnoses and identify rare or complex conditions that may be difficult to diagnose. Utilizing patient data, artificial neural network (ANN) models can be used to stratify patients into various risk categories of different diseases they are prone to. Based only on personal health data, one such ANN has been constructed to predict pancreatic cancer with a sensitivity of 80.7% and a specificity of 80.7% (Muhammad et al., 2019). In order to provide more specialized screening and risk management, the created ANN was also able to stratify people into groups with low, medium, and high cancer risk. Another population-based ML model has been shown to predict accurately a woman's 5-year risk of getting endometrial cancer based solely on her personal health data, without the use of invasive tests, imaging or genomic biomarkers (Hart et al., 2020).

Quite often there are rare conditions and syndromes that are over emphasized, and the treating consultant ends up doing extensive and often expensive workup unnecessarily. With AI, a physician would have better understanding of actual likelihood of such rare conditions to avoid unwarranted investigations. Also, with the help of AI, a wider range of potential diagnoses can be looked for in a shorter amount of time. This can help to reduce the time it takes to diagnose a patient and ensure that they receive appropriate treatment as quickly as possible. A few examples of this application of AI include Isabel DDx Companion and Glass AI (Glass, 2023; Isabel DDx Companion, 2023). LLMs like GPT-4 have also been shown to assist in the process of differential diagnosis (Nori et al., 2023). Given its exceptional capability for reasoning, it is perceivable that GPT-4 could be a regular tool in clinics and medical education in the near future.

### Pathology

Data processing and learning have become essential in advancing medicine, including the field of pathology and laboratory medicine. A growing area of expertise termed computational pathology uses modern digital communication networks and information integration to provide better-integrated solutions for whole-slide images, multi-omics data, and clinical informatics (Cui and Zhang, 2021). Computational pathology attempts to improve clinical workflow effectiveness, diagnostic quality, and patient-specific treatment regimens by utilizing digital pathology, which entails digitizing histopathology, immunohistochemistry, and cytology slides using whole-slide scanners (Cui and Zhang, 2021).

Various studies have demonstrated the effectiveness of AI in pathology. Deep neural network-based algorithms have been developed for accurate image classification and categorization of various neoplasms including prostate cancer, basal cell carcinoma, and breast malignancy (Campanella et al., 2019). Similarly, convolutional neural networks have been shown to outperform traditional machine learning classifiers in diagnosing prostate cancer using different imaging sources (Wildeboer et al., 2020). In the context of colorectal cancer, AI algorithms have been successful in classifying different types of colorectal polyps and predicting patient outcomes based on tissue samples (Korbar et al., 2017; Bychkov et al., 2018). Additionally, AI has shown promise in breast cancer diagnosis, biomarker assessment and sentinel lymph node analysis, leading to increased accuracy and efficiency in pathologists' diagnoses (Hamidinekoo et al., 2018; Robertson et al., 2018; Houssami et al., 2019). CNN techniques have alsodisplayed notable success in neuropathology immunohistochemistry (IHC), effectively classifying tauopathies according to p-tau lesions, categorizing Aβ lesions, and accurately quantifying alpha-synuclein burden from submandibular gland biopsies (Koga et al., 2021; Signaevsky et al., 2022; Wong et al., 2022).

Overall, AI's potential in computational pathology extends beyond morphological pattern detection. Its ability to integrate diverse clinical data enables it to contribute to various aspects of the clinical workflow, significantly improving medical outcomes. As AI algorithms continue to advance, the collaboration between AI and pathologists holds great promise for transforming pathology and delivering better patient care.

### Imaging

With the development of extremely precise computer vision technologies, there has been an upsurge in research into the subject of AI in clinical radiology. AI has various uses in the field, including assisted reporting, follow-up planning, data storage, data mining, and many others. It may also be used to acquire and process images. It has been used quite extensively in imaging in various fields ranging from neurology to oncology and applied to various imaging modalities, ranging from an X-Ray to Positron Emission Tomography (PET) scan. The major driving force behind this development was the availability of large datasets through RadImageNet, REFINE SPECT (REgistry of Fast Myocardial Perfusion Imaging with NExt generation SPECT), National Institutes of Health (NIH). Chest X-ray dataset and other imaging datasets (Clinical Center, 2017; Slomka et al., 2020; Mei et al., 2022). ML algorithms, DL networks, and other computational techniques can be trained on such large datasets of medical images to learn patterns and features that are characteristic of different diseases, structures, or abnormalities. Once trained, the AI algorithm can be applied to new, unseen images to automatically detect, classify, segment, or enhance them. The algorithm compares the features of the new images to the patterns it learned during training, and makes a prediction or decision based on the similarity between them. CNNs have paved way for widespread use of AI in imaging modalities. By applying a number of convolutional filters to the input data, such as images, CNNs are made to automatically learn and extract significant features. In many image-related applications, CNNs have attained state-of-the-art performance, revolutionizing the field of computer vision.

One of the significant applications of AI in radiology is the detection of lung diseases. DL algorithms have been used to detect malignant pulmonary nodules, pneumonia, lung fibrosis, tuberculosis and pleural effusions on chest radiographs (Nam et al., 2019; Kundu et al., 2021; Rajasenbagam et al., 2021; Huang et al., 2022; Li et al., 2022; Kazemzadeh et al., 2023). Breast cancer detection is another area where AI has made a substantial impact (Shen et al., 2019; Yoon and Kim, 2021). The currently available AI technology also allows for automated echocardiographic measurements including classifying severity of valvular heart diseases and identification of patients with coronary artery disease (Nedadur et al., 2022; Upton et al., 2022; Barry et al., 2023). In a study, a CNN-LSTM model was constructed to differentiate between the various etiologies of left ventricular hypertrophy. The DL algorithm's overall diagnostic accuracy was shown to be substantially greater than that of echocardiography experts (Hwang et al., 2022). The applications of AI echocardiography are way more than the examples mentioned and is rapidly expanding (Barry et al., 2023).

Ischemic stroke is an disease with high morbidity and mortality. The application of AI in stroke imaging has enabled early stroke diagnosis, automated calculations of Alberta stroke program early CT score (ASPECTS) score, detection of large vessel occlusion, evaluation of ischemic core and penumbra, and predicting functional outcomes after the stroke (Cui et al., 2022). AI has also been employed for detection and classification of neurological disorders such as Alzheimer's disease, multiple sclerosis, acute cerebral infarction and brain tumors using magnetic resonance imaging (MRI) data (Eshaghi et al., 2021; Frizzell et al., 2022; He et al., 2022; Ranjbarzadeh et al., 2023). Notably, AI systems have been demonstrated to perform better than radiology residents, general radiologists and neuroradiology fellows in generating top three accurate differential diagnosis on brain MRI (Rauschecker et al., 2020). Another use of AI in imaging apart from improving sensitivity and specificity is in reducing the amount of contrast agent used and duration of radiation exposure. SubtleGAD^TM^ is a DL model that can predict contrast enhanced images of MRI with only 10% of contrast agent dose. Structural similarity index measure (SSIM) between full dose and prediction models was 0.92 ± 0.02 using this model (Pasumarthi et al., 2021; SubtleGAD, 2023).

AI has also emerged as a transformative technology in the field of endoscopy, offering promising advancements with its sophisticated algorithms and machine learning capabilities like computer-aided detection (CADe) and diagnosis (CADx) of abnormalities, such as polyps and lesions, leading to improved accuracy and reduced miss rates (El Hajjar and Rey, 2020; Okagawa et al., 2022). AI can also assist in early diagnosis of gastric cancer as well as staging and estimation of depth of invasion (Kanesaka et al., 2018; Yoon et al., 2018). Optical biopsy, a novel application, allows real-time histology prediction based on endoscopic images, potentially mitigating the need for tissue sampling. Additionally, AI-driven colonoscope guidance aids in smoother insertion and loop prevention, benefiting patients and endoscopists alike. In video capsule endoscopy, AI algorithms aids in detecting small bowel bleeding, leading to quicker diagnosis of obscure gastrointestinal bleeding (Pan et al., 2011; Hassan and Haque, 2015). For IBD patients, AI can analyse colonoscopy images and predict mucosal inflammatory activity (Maeda et al., 2019). These applications highlight the potential of AI to revolutionize endoscopy by improving diagnostic accuracy, reducing procedural burden, and enhancing overall patient care.

### Therapeutics

AI has a pertinent role in disease management and medical therapeutics. As more evidence-based research is being conducted, newer data is made available for precise treatment plans and personalized intervention. Precision medicine involves using patient data, including genetic information, to develop personalized treatment plans (König et al., 2017). AI algorithms can analyse this data to predict patient outcomes and recommend tailored treatment plans, including medication options (Johnson et al., 2021). Data repositories have been created to facilitate the use of genomic data and deep phenotyping to aid in the practice of precision medicine (Nagai et al., 2017; Bycroft et al., 2018; Stark et al., 2019). Large amounts of genomic data can be analyzed by AI models to identify genetic variations that may be associated with specific diseases or conditions and develop personalized treatment plans based on an individual's genetic profile (Huang et al., 2018). Companies like Foundation Medicine specialize in genomic profiling of cancer patients to predict tumor behavior and response to therapy (FoundationOne Liquid CDx, 2023). In a recent phase III randomized controlled trial, a team from Genentech Inc. and Foundation Medicine Inc. used circulating tumor DNA (ctDNA) metrics to predict overall survival and pain-free survival patterns in patients with non-small-cell lung cancer (NSCLC), assisting in the early prediction trial outcomes (Assaf et al., 2023).

Clinical decision support systems (CDSS) are software applications that use AI algorithms to provide healthcare providers with real-time information and recommendations for patient care (Sutton et al., 2020). CDSS are typically integrated into electronic health record (EHR) systems and can provide a range of decision support functions, including clinical guidelines, health protocols, drug interaction alerts, clinical documentation, predictive analyses, and diagnostic and treatment recommendations based on patient-specific data and evidence-based guidelines (Sutton et al., 2020). For example, IBM Watson for Oncology (WFO), a software as a service (SaaS) developed by IBM Corporation (USA) with the help of top oncologists from Memorial Sloan Kettering Cancer Center (MSK), is a cognitive computing decision support system that provides evidence-based treatment recommendations for cancer patients (5725-W51 IBM Watson for Oncology, 2020). Studies have been conducted assessing the efficiency of WFO, and have reported high consistency between decisions made by WFO and multidisciplinary teams in most cancers, lower human-made error rate in chemotherapy drug regimen selection and significant improvement in work efficiency of doctors providing care to cancer patients (Jie et al., 2021).

AI is also being used to monitor patient health data in real-time, also known as remote patient monitoring (Shaik et al., 2023). For example, the start-up company Current Health has developed a wearable device that monitors patients' vital signs and uses AI to detect early warning signs of deterioration, which can help prevent hospital readmissions (Hospital at Home Powered by Current Health, 2023). AI-powered tools are also being used to monitor patient medication adherence, detecting when patients miss doses or stop taking medication (Babel et al., 2021).

### Prognosis and outcome prediction

One of the significant impacts of AI on healthcare is the ability to accurately predict disease prognosis. Prognosis is the prediction of the probable outcome of a disease, which is critical in healthcare, as it helps doctors make informed decisions about patient care, including treatment options and future health management (Rizzi, 1993).

One of the most widely used techniques is predictive modeling, which uses ML algorithms to analyse enormous amounts of medical data to find patterns and correlations that can forecast a patient's likelihood of contracting a specific disease or the likely course of that disease (readmissions, mortality, etc.). Through big data analytics, AI systems are able to develop predictive models that can identify patients who are at risk of developing certain diseases or conditions. One such venture is DeepMind, a subsidiary of Alphabet, that has an AI system that can predict the onset of acute kidney injury up to 48 h in advance using patient data (Using AI to give doctors a 48-hour head start on life-threatening illness, 2023). In order to estimate the death of dialysis patients while they are waiting for a kidney transplant, a method for survival prediction based on ML technique was recently proposed using sociodemographic data (Díez-Sanmartín et al., 2023). Studies have demonstrated the efficacy of ML models in predicting symptoms and mortality associated with COVID-19 using evidence that is readily available to patients as well as professionals (Jamshidi et al., 2021). As a result, both can assess the disease's severity quickly, enabling doctors to make better-informed healthcare decisions on hospitalization. Random forest ML models have also been validated for predicting 90-day readmission risk and cause at the time of discharge from index admissions for chronic obstructive pulmonary disease (COPD) patients (Bonomo et al., 2022). Recently, eXtreme Gradient Boosting (XGBoost) ML models trained on large COVID datasets have also been developed and validated to identify potential long COVID among all patients with COVID-19 with high accuracy (Pfaff et al., 2022).

To create more accurate and thorough prognostic models, AI may combine and analyse data from many sources, including omics (e.g., genomes, proteomics, and metabolomics) data, lifestyle factors, clinical measures, and demographics. For instance, the application of ML algorithms to multi-omics data has revealed novel chemicals such as cytokines, lipids, and metabolites as predictive biomarkers of severe and catastrophic outcomes after COVID-19 infection (Byeon et al., 2022). Recurrent neural network (RNN), a DL AI technology, has also been shown to predict 30 days of all-cause readmission following discharge from a heart failure admission (Allam et al., 2019). ML approaches have been used to predict COPD patients' 30-day hospital readmissions using data on their physical activity continually obtained via an accelerometer-based device (Verma and Lin, 2022).

In summary, AI shows great promise in revolutionizing the field of disease prognosis and outcome prediction. This can further assist clinicians and healthcare institutions to prioritize care and judiciously allocate resources.

### Research

The field of clinical research is constantly evolving, with new technologies and methodologies being developed to improve patient outcomes and advance medical knowledge. AI has the potential to transform the way clinical research is conducted, by enabling researchers to analyse large datasets, identify patterns and correlations, and make more accurate predictions about patient outcomes.

ML and DL have the ability to automatically identify patterns of meaning in huge datasets such as text, audio, or images, whereas NLP is capable of understanding and correlating evidence in spoken or written language. These capabilities can be used to automatically and continuously monitor patients throughout the trial as well as to correlate large and diverse datasets like EHRs, medical literature, and trial databases for better patient-trial matching and recruitment before a trial starts. By minimizing population heterogeneity, selecting patients who are more likely to have a measurable clinical outcome, and identifying a population more likely to respond to a treatment, AI models and methodologies can also be used to improve patient cohort selection (Harrer et al., 2019).

The process of discovering and studying newer therapies are often hindered by slow patient recruitment and selection processes AI can speed up the recruiting process, aid in the identification of possible participants, and ensure that the proper patients are chosen for the study, a phenomenon known as “electronic phenotyping” (Banda et al., 2018). Finally, by utilizing generative and prediction-based AI, ML, and reasoning techniques, AI can help with preclinical ligand discovery, drug-target testing, and defining lead compounds for clinical trials (Harrer et al., 2019; Bender and Cortés-Ciriano, 2021).

The integration of AI in clinical trials offers various strengths such as shorter patient recruitment times, less manpower for conducting studies and greater efficiency of dealing with data, but it also presents some notable weaknesses that need to be addressed (Khan et al., 2023). One significant challenge is the lack of data standardization, as diverse data sources and formats can hinder the seamless integration of data into AI algorithms, leading to potential biases and reduced accuracy. Additionally, the use of AI in clinical trials raises ethical and regulatory concerns, such as patient consent, data privacy, and potential biases in algorithmic decision-making. The interpretability and transparency of AI algorithms can also be problematic, as some models are considered “black boxes,” making it difficult to understand how they arrive at certain predictions. This lack of transparency can raise concerns for regulatory approval and may affect the trust of researchers and patients in AI-driven technologies. Moreover, implementing AI technologies in clinical trials may require substantial financial investment and specialized infrastructure, which may not be feasible for all research settings. Lastly, the limited generalizability of AI models trained on specific datasets may hinder their transferability to different patient populations or geographic regions, limiting their overall applicability in diverse healthcare settings. Addressing these weaknesses will be crucial to unlock the full potential of AI in clinical trials and to ensure its responsible and effective use in advancing medical research and patient care.

### AI in developing countries

While AI is making its way into a myriad of fields within the healthcare space (Table 2), a major sector of healthcare in developing nations remains underserved and in dire need of such tools. The quality and availability of healthcare services in developing countries significantly lags behind developed countries. This emphasizes on the requirement of technology to bridge this wide gap and ensure basic healthcare access in low and middle income countries (LMIC).

**Table 2:** AI innovations in clinical medicine.

**AI tool/software/company**	**Utility**
**Clinical evaluation**	
Suki (For Physicians, 2023)	• AI voice assistant for physicians to assist in taking notes and maintain electronic health records
VisualDx (2023)	• Clinical decision support tool that uses images and searchable clinical features to assist physicians in the diagnostic decision-making process
Isabel DDx Companion (2023)	• Diagnostic decision support tool that uses artificial intelligence and natural language processing to help clinicians improve their diagnostic accuracy • The system allows clinicians to enter a patient's symptoms, medical history, and other relevant information into the system, which then provides a list of potential diagnoses and related information to help the clinician make an accurate diagnosis
**Medical imaging**	
Radiology AI (2023)	• AI-powered software platform that analyzes X-ray, CT, and MRI images to flag critical findings and prioritize cases for radiologists • It can detect abnormalities such as intracranial hemorrhage, pulmonary embolism, and spine fractures, and has been shown to reduce turnaround times for urgent cases
qXR by qure.ai (qXR, 2023)	• Deep learning technology trained on over 4.2 million X-rays, aids in detecting comprehensive findings across lungs, pleura, mediastinum, bones, diaphragm, and heart on a chest X-ray • It segregates normal X-rays to reduce TAT for reporting and flags radiological signs of diseases like TB, lung cancer & heart failure
Viz.ai (2023)	• A company that offers AI-powered solutions in the field of healthcare imaging • Viz.ai LVO uses deep learning algorithms to analyze CT scans of the brain and detect large vessel occlusions (LVOs) in patients with suspected stroke with high accuracy and also sends an automated alert to the stroke team, allowing them to quickly initiate treatment • Viz.ai CTP analyses CT perfusion images and provide automated analysis of blood flow, volume, and permeability. Similarly, the company has other AI-based softwares in other fields of imaging
Arterys (Medical Imaging Cloud AI for Radiology, 2023)	• AI-based medical imaging analytics company that offers a cloud-based platform for analyzing CT scans, X-rays, Cardiac MRI and Digital Breast Tomosynthesis
**Therapeutics**	
AiCure (Patient Connect, 2023)	• Mobile App to assist in monitoring patient medication adherence in real-time using computer vison
IBM watson for oncology (5725-W51 IBM Watson for Oncology, 2020)	• Cognitive computing decision support system that provides evidence-based treatment recommendations for cancer patients
Lexicomp (2023)	• Clinical decision support tool and drug information database that provides healthcare professionals with accurate, up-to-date information on drugs, dosages, drug interactions, and clinical guidelines
**Research**	
Deep 6 AI (Home—Deep6.ai, 2023)	• A clinical trial recruitment platform that uses AI to identify eligible patients for clinical trials. The platform analyzes electronic health records (EHRs) to find patients who meet the inclusion and exclusion criteria for a given trial, and then matches them with available trials
Medidata AI (2023)	• A clinical trial management platform that uses AI to automate various aspects of the trial process, such as data capture and analysis • The platform uses natural language processing (NLP) to extract data from various sources, such as electronic health records (EHRs) and medical imaging data, and then analyzes the data to identify trends and patterns

In most developing nations, healthcare workers are overburdened and face time constraints. AI-powered systems can automate many tasks, such as data entry and patient scheduling, which can free up healthcare providers' time and improve patient care. In rural areas and remote regions where the population lacks access to proper infrastructure and healthcare professionals, AI can fill the gap through telemedicine (Kuziemsky et al., 2019; Alami et al., 2020). To attain health goals, mHealth uses wireless and mobile technologies. Mobile phone usage has grown rapidly in low-income nations, opening up an array of opportunities for employing such technologies to aid in health initiatives. AI solutions based on smartphones offer a potential remedy for screening underserved and under-resourced populations, enabling quick treatment, halting illness progression, and lowering mortality and morbidity (Alami et al., 2020). A smartphone app created at Emory University, for instance, can evaluate pictures of fingernail beds to calculate hemoglobin levels (Mannino et al., 2018). In a pilot trial, the application's sensitivity for detecting anemia was 97%. Machine-learning algorithms have been developed by a number of research collaborates to identify skin disorders in images recorded by smartphones (Pangti et al., 2021; Ouellette and Rao, 2022). A deep-learning technology developed by Stanford University (Stanford, CA, USA) researchers proved capable to characterize skin cancer with performance at par with certified dermatologists when evaluating clinical images (Esteva et al., 2017). Similar to this, smartphone applications have been developed with sensitivities and specificities of over 90% to detect dermatitis and psoriasis. These techniques could significantly broaden access to routine skin disease screenings and make it feasible for front-line staff to detect these conditions promptly (Chan et al., 2020). Smartphone applications can also accurately identify Parkinson's disease by analyzing patient voice samples using machine-learning algorithms (Singh and Xu, 2020). Other AI tools are also being used in developing nations to increase remote access to quality care. An excellent example of this is the use of deep learning and neural networks in remote screening for early changes in diabetic retinopathy (DR), so that patients can be diagnosed and referred to higher centers for management much earlier as compared to diagnosis with conventional screening methods. This application delivered via a smartphone device has been shown to have a high sensitivity and specificity in detecting DR for early referral to an ophthalmologist (Sosale et al., 2020). Another major development for DR screening is the DL system (Inception-V3), which has a high sensitivity (≥96%) and specificity (≥93%) in detecting DR. It was tested and validated by Google AI using a large training dataset of 128,175 and two separate publicly available datasets (Gulshan et al., 2016, 2019). ML models are also being trained to identify newborns with risk of birth asphyxia (Onu et al., 2017; Sachin et al., 2017; Darsareh et al., 2023). Given the high neonatal mortality in low and middle income countries, this could prove to be a revolutionary application of AI in such circumstances.

The role of AI in pandemics is also prominent, as it can be used to predict and mitigate the spread of infectious diseases. For example, AI-powered systems can analyse data from social media and other sources to identify potential disease outbreaks, which can enable early interventions to prevent the spread of the disease, assist in source identification, hotspot detection and, tracking and forecasting (Zeng et al., 2021; Brownstein et al., 2023). Various AI models have been employed to detect infectious disease outbreaks such as dengue, zika virus, influenza etc. (Akhtar et al., 2019; Anno et al., 2019; Zhu et al., 2019). Similarly, AI-powered contact tracing systems can help track and isolate individuals who may have been exposed to a disease (Saba and Elsheikh, 2020; Abbar and Mokbel, 2021; Wahid et al., 2023). The recent COVID-19 pandemic illustrated all too well the severe shortage of qualified medical professionals and also the inherent risks of infection due to person-to-person contact, culminating into a public health crisis. AI emerged as an ally in our fight against the pandemic by enhancing screening, treatment, contact tracing, prediction and forecasting and, drug and vaccine development (Lalmuanawma et al., 2020). Multiple ML and DL models were also developed during the pandemic to assist in triaging of patients and prioritize healthcare to the most needy (Liang et al., 2020; Wang et al., 2020; Park et al., 2022; Rajendran et al., 2022).

Finally, AI can play an important role in national database creation (Lauricella and Pêgo-Fernandes, 2022). In many developing nations, there is a lack of reliable data on healthcare. By ensuring that vital information is accessible for making informed policy and programme decisions, health informatics can aid in the shaping of public health projects. Health informatics relies heavily on EMRs, which are digital versions of patient and population health data. In an era of networked computers, their use has increased significantly in low-resource settings, which has increased the potential applications of AI to enhance public health decision-making and informatics. To make data entry quicker, more accurate, and easily verifiable, health records can be digitalized utilizing already available AI tools like optical character recognition, speech-to-text, and other NLP techniques. Greater linkages and record deduplication can be made possible by more advanced functions, ensuring an uninterrupted continuum of care. Many developing nations are currently using cloud-based EMR systems, with OpenMRS serving as one example (Verma et al., 2021). The system has been used in fields of maternal and child health as well as HIV management and has been found to increase the completeness of data collected and close critical gaps in care (Haskew et al., 2015a,b).

AI-based solutions can efficiently bring advanced, personalized expertise to the most far-flung patients and enable targeted, patient-centered healthcare delivery. The deployment of AI conserves human resources and ensures equal access to healthcare for people who reside away from major cities. Additionally, it can reduce the cost of basic medical care for individuals who cannot afford to visit a real doctor for treatment.

### AI in healthcare: the way forward

The research and application of AI in healthcare encounter a variety of difficulties, such as ethical dilemmas, legal issues, security concerns, and public acceptance (Cath, 2018). Informed consent, privacy, data protection, ownership, objectivity, and transparency are major issues in data ethics (Kayaalp, 2018). Patient rights and data use are questioned by the ownership of personal health data. Another key ethical problem is unfairness based on by prejudiced data, which can be generated by biases that stem from socioeconomic, racial, or gender reasons (Rajkomar et al., 2018). Healthcare disparities and ethical issues including biased resource allocation may result from it. Human elements such as mistakes made by developers or doctors could threaten patient health, which adds to concerns about AI security. Legal frameworks for DNA transmission, storage, and collecting ought to be strengthened. The application and standardization of AI-based healthcare solutions are made more difficult by the lack of globally established standards and regulations for AI in medicine. Finally, patients may place more trust in doctors than in AI-based diagnoses, which could pose problems with both trust and accountability.

The following steps can be taken to address the challenges mentioned earlier and to ensure smooth integration of AI in healthcare:

- Preliminary algorithms must be developed and tested for stability and safety before being applied in small-scale clinical settings. These algorithms should be optimized through experience in studies and eventually applied in large-scale clinical settings.
- By integrating blockchain and big data technologies, high-quality datasets can be securely stored and maintained.
- To manage the entire procedure and provide complaint channels for issues to be addressed and resolved quickly, a separate supervisory department must be established.
- An independent ethics committee must be established to oversee the entire AI development process, addressing ethical issues in data, resource allocation, and practice.
- Patients must retain ownership of their medical data, while the right to access the data for healthcare improvement purposes can be granted to healthcare providers or government entities.
- To ensure fairness, marginalized populations must be considered, and equal outcomes, performance, and allocation must be prioritized.
- A comprehensive legal system must be established to regulate every step of AI integration into healthcare. The system must be flexible, guiding AI development without hindering it.
- Doctors must remain skeptical of AI-generated information and make informed decisions based on patients' specific circumstances.
- AI products must be user-friendly, reliable, and stable. Feedback from users should be used to improve product performance and functionality.
- Increasing public exposure to AI through offline experience stores, social media, and live broadcast platforms can foster greater acceptance and trust in AI-based healthcare technologies.

As we conclude this review, its necessary to mention ‘Friedman’s fundamental theorem of informatics' which posits that the combination of human intelligence and AI is superior to human intelligence alone (Friedman, 2009). By integrating AI into our decision-making processes, we can leverage its strengths, such as processing large volumes of data, pattern recognition, and unbiased analysis, while also harnessing the creativity, empathy, and contextual understanding that humans bring to the table. The theorem suggests that a synergistic partnership between humans and AI will lead to better outcomes than relying solely on either entity. The integration of AI in healthcare is not meant to replace doctors, but rather to reshape their roles and augment their capabilities. In domains where challenges and complexities abound, such as healthcare, the integration of AI has the potential to improve diagnostics, treatments, and overall care, while still emphasizing the crucial role of human touch and emotional intelligence. In the era of AI, various industries are increasingly embracing this technology, and the medical field is no exception. This collaboration between humans and AI has the power to drive innovation, enhance decision-making, and foster a more informed and adaptive workforce, ultimately benefitting society as a whole.

## Conclusion

In conclusion, AI has demonstrated significant potential in shaping the future of clinical medicine. From enhancing history taking and clinical examination to streamlining imaging, therapeutics, prognosis and research, AI has made substantial strides in various aspects of patient care. Additionally, AI's contributions to healthcare in developing nations have bridged disparities and improved access to quality care. As AI technologies continue to evolve, it is essential for the medical community to embrace this innovation, ensuring ethical and responsible applications to optimize patient outcomes and promote global health equity.

## Author contributions

Conceptualization: AP, GK, and SS. Writing—original draft preparation: AP, GK, SS, SG, and SA. Writing—review and editing: AP, MP, and MD. Supervision: MP and MD. All authors contributed to the article and approved the submitted version.
